# L’apport de la radio-chimiothérapie concomittante dans la prise en charge du carcinome indifférencié du nasopharynx de l’adulte

**DOI:** 10.11604/pamj.2018.31.98.14951

**Published:** 2018-10-10

**Authors:** Zenab Alami, Touria Bouhafa, Abderrahmane Elmazghi, Khalid Hassouni

**Affiliations:** 1 Service de Radiothérapie, CHU Hassan II, Faculté de Médecine et de Pharmacie de Fès, Maroc

**Keywords:** Nasopharynx, cancer, radiothérapie, chimiothérapie, pronostic, Nasopharynx, cancer, radiotherapy, chemotherapy, prognosis

## Abstract

L'objectif de l'étude et d'Analyser les caractéristiques épidémiologiques, cliniques, thérapeutiques et évolutives du carcinome indifférencié du nasopharynx de l'adulte. Il s'agit d'une étude cohorte rétrospective portant sur 163 patients âgés de 17 ans et plus, traités pour un carcinome indifférencié du nasopharynx non métastatique. L'âge moyen des patients était de 46,5ans, avec un sexe-ratio de 1,7. 35,57% des patients étaient atteints de tumeurs localement évoluées (T3-T4) et 52,27% avec une atteinte ganglionnaire régionale avancée (N2-N3). Sur le plan thérapeutique une chimiothérapie neoadjuvante a été réalisée chez 77% des patients et 93,8% des patients ont bénéficié d'une radio-chimiothérapie concomitante. Après un recul moyen de 40,8 mois la survie globale était de 92,9% et la survie sans récidive (SSR) était de 78,9%. L'analyse de la survie sans récidive SSR en fonction des différents facteurs pronostiques a montré une différence statistiquement significative pour l'atteint ganglionnaire avec des taux de SSR à trois ans de 88%, 82,6%, 80,8% et 61,5% en cas de tumeur classée N0, N1, N2 et N3 respectivement (p = 0,02). Le cancer du nasopharynx est une maladie complexe, mais des progrès ont été accomplis grâce à des percées en radiothérapie et en biologie moléculaire. La radio-chimiothérapie concomitante représente le standard thérapeutique des stades cliniques supérieur ou égal à T2, ou supérieur ou égal à N1. Les techniques innovantes d'irradiation semblent prometteuses et pourraient pallier aux problèmes de toxicité tardive tout en assurant un excellent taux de contrôle local.

## Abstract (en)

This study aimed to investigate the epidemiological, clinical, therapeutic and evolutionary features of undifferentiated carcinoma of the nasopharynx in adults. We conducted a retrospective cohort study of 163 patients aged 17 years old and over, treated for non metastatic undifferentiated carcinoma of the nasopharynx. The average age of our patients was 46,5 years, with a sex-ratio of 1.7; 35.57% of patients had locally advanced tumors (T3-T4) and 52.27% had advanced regional lymph nodes involvement (N2-N3). Neoadjuvante chemotherapy was performed in 77% of patients and 93.8% of patients underwent concomitant radiochemotherapy. After a mean follow-up interval of 40.8 months overall survival was 92.9% and relapse-free survival (RFS) was 78.9%. Relapse-free survival was caculated according to different prognostic factors, revealing a statistically significant difference based on lymph nodes involvement; three-year RFS rates were 88%, 82.6%, 80.8% and 61.5% in patients with tumor classified as N0, N1, N2 and N3, respectively (p = 0.02). Nasopharyngeal cancer is a complex disease, but progress has been made thanks to advances in radiotherapy and molecular biology. Concomitant radiochemotherapy is the therapeutic standard for patients with clinical stage greater than or equal to T2, or greater than or equal to N1. The innovative techniques in radiation therapy appear promising and they could reduce late toxicity while ensuring an excellent local control rate.

## Introduction

Le carcinome indifférencié du nasopharynx (ou l'UCNT du cavum) représente la forme histologique la plus fréquente des cancers du cavum aux pays du maghreb, il diffère des autres carcinomes épidermoïdes de la tête et du cou par son histologie indifférenciée caractéristique, son épidémiologie sans rapport avec l´alcool et le tabac et sa relation constante avec le virus d´Epstein-Barr (EBV). La radiothérapie reste le principal traitement des UCNT, et les progrès récents permettent maintenant d´envisager une meilleure probabilité de contrôle tumoral tout en limitant la morbidité (radiothérapie conformtaionnelle par modulation d´intensité). Parmi les patients présentant une maladie localement évoluée, la chimiothérapie associée à la radiothérapie a permis d´augmenter la survie sans récidive dans plusieurs essais.

## Méthodes

**Patients**: Il s'agit d'une étude de type cohorte rétrospective portant sur 163 patients, traités pour un UCNT du cavum non métastatique, au Service de Radiothérapie du CHU Hassan II de Fès. On a inclus tous les patients âgés de 17 ans et plus, diagnostiqués entre Janvier 2012 et Décembre 2014. De cette étude sont exclus: les patients âgés de moins de 17 ans, les patients ayant un diagnostic histologique autre que l'UCNT du cavum, les patients avec un cancers du cavum métastatique, ainsi que les patients perdu de vue avant le début du traitement. Tous nos patients ont bénéficié d´une cavoscopie avec un prélèvement biopsique pour examen anatomopathologique. Le bilan d'extension Locorégional a compris une TDM ou IRM du cavum et du cou. Le bilan d'extension à distance a compris une TDM thoracique et une échographie abdominale + /- une scintigraphie osseuse en fonction du contexte clinique de chaque patient.

**Traitement**: La chimiothérapie d'induction a été indiquée en cas d'atteinte ganglionnaire et a consisté en trois cycles à base de cisplatine suivi d'une radiothérapie locorégionale. La radiothérapie a été réalisée selon une technique conformationnelle tridimensionnelle (RC3D). La dose a été délivrée en deux temps: 70 Gy en 35 fractions sur le PTV Haut Risque et 50Gy en 25 fractions sur le PTV bas risque. Un contrôle de positionnement a été réalisé avant le début du traitement puis de façon hebdomadaire. L'évaluation des patients était prospective à raison d'une consultation hebdomadaire durant la radiothérapie. Une mise en état de la cavité buccale avec des extractions dentaires et une confection de gouttière dentaire en vue d'une prophylaxie fluorée ont été systématiquement effectuées. La chimiothérapie concomitante à base de sel de platine a été indiquée en cas de tumeur T3 ou T4 ou en cas d'attente ganglionnaire. Pour Les patients ayant eu une chimiothérapie néoadjuvante, un examen clinique, associé ou non à une imagerie du cavum ont été réalisés avant de débuter l'irradiation.

**Évaluation de la réponse et surveillance**: Après la fin du traitement, l'évaluation des patients était prospective à raison d'une consultation trimestrielle pendant 2 ans puis semestrielle pendant 3 ans. Au delà de cinq ans le suivi était annuel. Une endoscopie du cavum a été demandé à la première consultation de surveillance puis annuellement. Une scanographie et/ou IRM du cavum a été demandée à 6mois du traitement puis annuellement.

**Étude statistique**: Les probabilités de survie ont été estimées en utilisant la méthode de Kaplan Meier. Pour déterminer l'impact de chaque variable sur la survie globale et la survie sans récidive, les caractéristiques des patients et de la tumeur ont été individuellement analysées en utilisant le test Log rank. Toutes les analyses statistiques ont été réalisés par le logiciel SPSS (Statistical Package for the Social Sciences) au laboratoire d'épidémiologie du CHU Hassan II de Fès.

## Résultats

**Caractéristiques des patients**: 163 patients ont été inclus dans l'étude. L'âge de nos patients varie entre 17 ans et 88 ans, avec un âge médian de 47 ans, la moyenne d'âge est 46,5 ans, la tranche d'âge la plus touchée est de 41 à 60 ans, elle représente 47.2 % des cas, avec un sexe-ratio de 1.7 et une prédominance masculine. Le délai moyen de consultation était de 8 mois variant de 1 mois à 24 mois, avec une médiane de 7 mois. La symptomatologie clinique était dominée par un syndrome ganglionnaire qui était présents chez 74,8 % des patients. Le syndrome rhynologique était présent chez 60,1% des patients, le syndorme othologique chez 55,15 % des cas, et le syndrome neurologique chez 30,6 %. Selon la claasification TNM (UICC-AJCC) sataging system édition 2010), 35,57 % des patients étaient atteints de tumeurs localement évoluées (T3-T4), et 52,27% avec une atteinte ganglionnaire régionale avancée (N2-N3) (Tableau 1).

**Tableau 1:** Les caractéristiques des patients

Paramètres	N(%)
**Sexe**	
Homme	103 (63,19%)
Femme	60 (36,8%)
**Age**	
moyen	46,5ans
**Présentation clinique**	
Syndrome ganglionnaire	122 (74,8%)
Signes rhinologiques	98 (60,1%)
Signes otologiques	91 (55,15%)
Signes neurologiques	50 (30,6%)
**Type histologique**	
Type histologique: UCNT	163 (100%)
**Classification T**	
T1	29 (17,8%)
T2	76 (46,6%)
T3	33 (20,24%)
T4	25 (15,33%)
**N classification**	
N0	25 (15,3%)
N1	52 (31,9%)
N2	73 (44,7%)
N3	13 (7,97%)
**M classification**	
M0	163
M1	0

**Figure 1:**
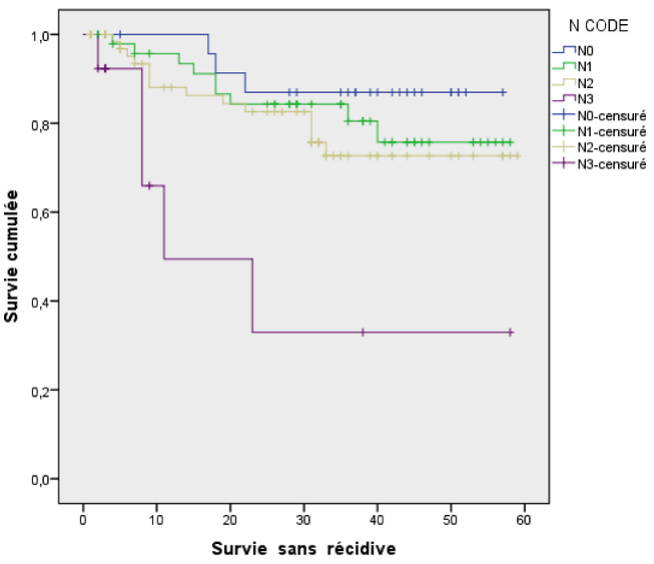
Survie sans récidive en fonction de l’atteinte ganglionnaire

**Résultats thérapeutiques et évolution**: Une chimiothérapie neoadjuvante a été réalisée chez 77 % des patients, et 93,8 % des patients ont bénéficié d'une chimiothérapie concomitante à la radiothérapie. Le délai moyen entre la fin de la chimiothérapie néoadjuvante et le début de la Radiothérapie était de 23 jours (14-33jours). L'étalement moyen de la radiothérapie était de 57 jours (47-70 jours). Après un recul moyen de 40,8 mois, calculé de la date de fin de traitement la survie globale était de 92,9% et la survie sans récidive était de 78,9%. Parmi les patients ayant présentés une récidive, 19 étaient en récidive métastatique et 13 étaient en récidive locorégionale. A noter que 28 de nos patients ont été perdu de vue après la fin du traitement. L'analyse de la survie sans récidive SSR en fonction des différents facteurs pronostiques a montré une différence statistiquement significative pour l'atteint ganglionnaire avec des taux de SSR à trois ans de 88 %, 82,6 %, 80,8 % et 61,5 % respectivement en cas de tumeur classée N0, N1, N2 et N3 (p = 0,02) (Figure 1). Pour les autres facteurs pronostiques étudiés, la différence n'était pas statistiquement significative (Tableau 2).

**Tableau 2:** Taux de survie sans récidive à trois ans en fonction des facteurs pronostiques

Paramètres	Survie sans récidive à 3 ans (%)	p-Value
**Age**		
<60 ans	81,2	0,577
>60 ans	80	
**Sexe**		
Homme	82.5	0,69
Femme	78,3	
**Tumeur**		
T1-T2	82,8	0,066
T3-T4	77,5	
**Adénopathie**		
N0	88	0,02
N1	82,6	
N2	80,8	
N3	61,5	

## Discussion

Selon l'organisation mondiale de la santé [1] les carcinomes nasopharyngés peuvent être divisés en 3 types comme suite: Type 1) carcinome épidermoide, qui survient plus souvent à l'âge adulte, Type 2) carcinome non Kératinisant et le Type 3) le carcinome indifférencié. Dans notre étude la plus part des patients étaient dans la 5^ème^ décennie de vie. Il n y avait pas de distribution bimodale. La répartition géographique très contrastée de ce type de cancer représente une des Caractéristiques de la maladie. En effet, à l´échelle mondiale, il existe trois zones : une zone à très Haute fréquence avec la Chine du Sud (Canton), où l´incidence est de 30 à 80/100.000/an. Une zone à fréquence intermédiaire (8 à 12/100.000/an) avec Taiwan, le Vietnam, la Thaïlande, la Malaisie, les Philippines, les Caraïbes, le bassin méditerranéen (Maghreb et Moyen-Orient), l'Alaska et le Groenland. Et enfin une zone à fréquence faible en Europe et aux Etats-Unis (0,5 à 2/100 000/an). Cette affection est plus fréquemment observée chez les sujets après 50 ans. Son sexe ratio est de deux, trois hommes pour une femme. Le rôle du tabac est controversé mais reste évoqué dans une méta-analyse publiée en 2013, qui retenait une augmentation du risque de 60 % pour les fumeurs, et ce même pour les formes indifférenciées [2]. Le tabac et l'alcool sont les facteurs de risque majeurs dans les cancers des voies aérodigestives supérieur. Les carcinomes indifférenciés du nasopharynx sont cependant moins affectés par ses facteurs qui ne sont pas considérés des facteurs étiologiques dans ces cas. La radiotherapie en combiairson à la chimiothérapie neoadjuvante ou concomittante sont devenu actuellement l'arme thérapeutique majeur dans la prise en charge des carcinome nasopharyngés.

**La radiothérapie seule**: Le nasopharynx n'est pas facile à aborder chirurgicalement en raison de son anatomie complexe. L´intervention chirurgicale primaire a été abondonnée dans les années 1950, et la plupart des traitements consistent en une chimioradiothérapie concomitante. De nombreuses études ont déjà montré que la radiothérapie seule avait des taux de survie à 10 ans de 34%, 37% et 43% [3-5]. L'étude chinoise avait le taux de survie à dix ans le plus élevé. Cependant dans cette série il y avait beaucoup de patient avec une maladie de stade précoce (39% : N0), et peu de patients ayant une histologie de type kératinisante qui est de mauvais pronostic (0,3%). Les études américaines et européennes avaient un pourcentage plus important de patients avec une histologie de type Keratinisante. Dans l'étude de Sanguineti G *et al*. [3] les auteurs ont montré que le stade T avancé, le carcinome épidermoide, et l'atteinte des nerfs craniens prédisent un mauvais pronostic si la Radiothérapie est utilisée seule. Un Bon control tumoral peut être obtenu par radiothérapie seule pour les maladies de stade précoce, mais les traitements combinés, tels que la chimio-radiothérapie, sont nécessaires pour les cancers avancés. Des améliorations significatives ont été faites dans le traitement de la maladie de stade avancé avec les protocoles associant la chimiothérapie et la radiothérapie et les nouvelles techniques d'irradiation, toutefois l'utilisation de la radiothérapie conformationnelle par modulation d'intensité (RCMI) a permis de délivrés des doses plus élevées de rayonnement à la tumeur ainsi qu'une réduction significative des toxicités associées, rendant ainsi la RCMI la technique d'irradiation de choix dans les deux stades précoce et avancé [6, 7].

**L'association radio chimiothérapie**: Plusieurs groupes ont conduit des essais phase III spécifiques aux tumeurs primitives du nasopharynx comparant la chimiotéhrapie associé à la radiothérapie à la radiothérapie seule [9-15] (Tableau 3). L'étude de l'intergroupe 0099, à montré une amélioration importante dans les taux de survie avec le traitement combiné [8]. Les patients ont été randomisés entre recevoir une chimio radiothérapie ou une radiothérapie seule. Les patients ont reçu une radiotéhrapie standard de 70Gy au volume tumoral avec fractionnement classique de 1,8Gy à 2Gy par jour. La chimiothérapie ajouté était cis-platine 100mg/m^2^) J1, J22 et J43 d'irradiation ainsi que Cisplatine (80mg/m^2^) et 5-Fluorouracil (5FU) (1000mg/m^2^)) toutes les 3 semaines pendants 3 cycles après la fin de la radiothérapie. Comme démontré dans Tableau 3, il y avait une amélioration statistiquement significative en survie Globale et survie sans récidive dans le bras radio-chimiothérapie. Des études chez des patients chinois [16] ont montré des toxicités sévères ou des taux élevés de métastases à distance en dehors du champ irradié. D'autres études ont suggérés que le mauvais résultat dans le bras Radiothérapie seule était secondaire à la technique de radiotéhrapie [17]. Néanmoins, le résultat de cet essai a changé la conduite thérapeutique en matière de prise en charge des cancers du nasopharynx. Par conséquence, l'amélioration importante dans les résultats du traitement combiné a limité l'utilisation de la radiothérapie seule aux patients avec une maladie T1. Les essais conduits dans les zones endémiques, ont confirmé l'avantage de la chimiotéhrapie à base de cisplatine en association à la radiothérapie. Une métaanalyse de 7 essais phase III comparant la chimio radiothérapie concomittante versus radiothérapie seule, incluant 1608 patients de régions endémiques, a démontré un avantage en survie globale en faveur du régime concomittant avec un risque relatif de 0,74 à 5ans [18]. Cet avantage en survie est moins important que celui observé dans les essais réalisés dans des régions non-endémiques. Dans notres série qui a été réalisé dans une popultation considérée comme endémique, les résultats de la radiochimioéthérapie sont concordants avec ceux de la littérature avec une survie Globale à 3 ans de 92,9% et une survie sans récidive de 78,9% avec comme facteur majeur de mauvais pronostic, l'atteinte ganglionnaire.

**Tableau 3:** Résultats thérapeutiques de la radio chimiothérapie concomitante

Etude	Nombre de patients	Stade	Randomization	Survie Sans Récidive (%) à 5 ans	Survie Globale (%) 5 ans
Intergroup 0099, 1998. [8]	150	Stade IV: T4N0-1; TN2-3M0	70 Gy/7-8 sem avec cisplatin, suivi par 3 cycles de cisplatin/ 5- fluorouracil	58	67
Chan *et al*. 2002; [9] Chan *et al*. 2005 [10]	350	N2-3 ou tout N>4 cm	66 Gy/6.5 sem 10-20 Gy boost avec cisplatine	60	70
Lin *et al*. 2003 [11]	284	III-IV (M0)	70-74 Gy/6-7sem avec cisplatin/5- fluorouracil x 2	72	72
Wee *et al*. 2005 [12]	221	Stade III ou IV;	70 Gy/7sem avc cisplatin, suivi par 3 cycles de cisplatin /5- fluorouracil	73 (3 ans)	80 (3 ans)
Lee *et al*. 2005 [13]	348	T1-4N2-3M0;	>66 Gy/7-8 sem avec cisplatin, suivi par 3 cycles de cisplatin/5-fluorouracil	72	78
Lee *et al*. 2011 [14]	370	Stade III-IVB	>60Gy avec cisplatine, suivi par 3 cycles de cisplatin/ 5-fluorouracil	-	72
Dechaphunkul *et al*. 2011 [15]	50	T3 ou N1 ≥ 3 cm ou ≥ N2	66 à 70Gy carboplatine et carboplatine/ 5- fluorouracil	72,7 (3 ans)	89,7 (3 ans)
Notre série	163	Stade I-IVB	70Gy/7-8sem avec Cisplatine +/- 2 à3 cycles de cisplatine/ 5-fluorouracil en neadjuvant	78,9 (3 ans)	92,9 (3 ans)

## Conclusion

Le cancer du nasopharynx est une maladie complexe, mais des progrès ont été accomplis grâce à des percées en radiothérapie et en biologie moléculaire. La maladie à un stade précoce peut répondre à la radiothérapie seule avec un bon contrôle local. Pour un stade supérieur ou égal à T2, ou supérieur ou égal à N1, la chimio-radiothérapie concomittante est préférée. Les techniques innovantes d'irradiation semblent prometteuses et pourraient pallier aux problèmes de toxicité tardive tout en assurant un excellent taux de contrôle local.

### Etat des connaissances actuelles sur le sujet

- Le Maroc constitue une zone à fréquence intermédiaire du cancer du nasopharynx;
- La Radio-chimiothérapie concomitante représente l'arme thérapeutique majeur dans la prise en charge de l'UCNT du nasopharynx;
- Les facteurs pronostics sont dominés par le stade initial de la maladie y compris la taille initiale de la tumeur (T), ainsi que l'atteinte ganglionnaire (N).

### Contribution de notre étude à la connaissance

- A notre connaissance, aucune étude élaborée sur ce sujet n'est disponible dans la région de Fès;
- Les résultats thérapeutiques de l'association radio chimiothérapie sont concordant avec les résultats de la littérature notamment en matière de survie Globale et de survie sans récidive;
- L'atteinte ganglionnaire (Stade N3) est un facteur de mauvais pronostic.

## Conflits d’intérêts

Les auteurs ne déclarent aucun conflits d'intérêts.

## References

[1] Barnes L, Eveson JW, Reichart P, Sidransky D (2005). World Health Organisation classification of tumours: pathology and genetics of head and neck tumours.

[2] Xue WQ, Qin HD, Ruan HL, Shugart YY, Jia WH (2013). Quantitative association of tobacco smoking with the risk of nasopharyngeal carcinoma: a comprehensive meta-analysis of studies conducted between 1979 and 2011. Am J Epidemiol.

[3] Sanguineti G, Geara FB, Garden AS (1997). Carcinoma of the nasopharynx treated by radiotherapy alone: determinants of local and regional control. Int J Radiat Oncol Biol Phys.

[4] Johansen LV, Mestre M, Overgaard J (1992). Carcinoma of the nasopharynx: analysis of treatment results in 167 consecutively admitted patients. Head Neck.

[5] Lee AW, Poon YF, Foo W (1992). Retrospective analysis of 5,037 patients with naso- pharyngeal carcinoma treated during 1976-1985: overall survival and patterns of failure. Int J Radiat Oncol Biol Phys.

[6] Lee N, Xia P, Quivey JM (2002). Intensity-modulated radiotherapy in the treatment of nasopharyngeal carcinoma: an update of the UCSF experience. Int J Radiat Oncol Biol Phys.

[7] Pow EH, Kwong DL, McMillan AS (2006). Xerostomia and quality of life after intensity-modulated radiotherapy versus conventional radiotherapy for early-stage nasopharyngeal carcinoma: initial report on a randomized controlled clinical trial. Int J Radiat Oncol Biol Phys.

[8] Al-Sarraf M, LeBlanc M, Giri PG (1998). Chemoradiotherapy versus radiotherapy in patients with advanced nasopharyngeal cancer: phase III randomized intergroup study 0099. J Clin Oncol.

[9] Chan AT, Teo PM, Ngan RK (2002). Concurrent chemotherapy-radiotherapy compared with radiotherapy alone in locoregionally advanced nasopharyngeal carcinoma: progression-free survival analysis of a phase III randomized trial. J Clin Oncol.

[10] Chan AT, Leung SF, Ngan RK (2005). Overall survival after concurrent cisplatin- radiotherapy compared with radiotherapy alone in locoregionally advanced naso- pharyngeal carcinoma. J Natl Cancer Inst.

[11] Lin JC, Jan JS, Hsu CY (2003). Phase III study of concurrent chemoradiotherapy versus radiotherapy alone for advanced nasopharyngeal carcinoma: positive effect on overall and progression-free survival. J Clin Oncol.

[12] Wee J, Tan EH, Tai BC (2005). Randomized trial of radiotherapy versus concurrent chemoradiotherapy followed by adjuvant chemotherapy in patients with american joint committee on Cancer/International union against cancer stage III and IV nasopharyngeal cancer of the endemic variety. J Clin Oncol.

[13] Lee AW, Lau WH, Tung SY (2005). Preliminary results of a randomized study on therapeutic gain by concurrent chemotherapy for regionally-advanced nasopha- ryngeal carcinoma: NPC-9901 trial by the hong kong nasopharyngeal cancer study group. J Clin Oncol.

[14] Lee AW, Tung SY, Ngan RK, Chappel lR, Chua DT, Lu TX (2011). Factors contributing to the efficacy of concurrent-adjuvant chemotherapy for locoregionally advanced nasopharyngeal carcinoma: Combined analyses of NPC-9901 and NPC-9902. Eur J Cancer.

[15] Dechaphunkul T, Pruegsanusak K, Sangthawan D, Sunpaweravong P (2011). Con- current chemoradiotherapy with carboplatin followed by carboplatin and 5-fluorouracil in locally advanced nasopharyngeal carcinoma. Head Neck Oncol.

[16] Chua DT, Sham JS, Au GK (2002). Concomitant chemoirradiation for stage III-IV nasopharyngeal carcinoma in chinese patients: results of a matched cohort analysis. Int J Radiat Oncol Biol Phys.

[17] Chow E, Payne D, O'Sullivan B (2002). Radiotherapy alone in patients with advanced nasopharyngeal cancer: comparison with an intergroup study is combined modality treatment really necessary?. Radiother Oncol.

[18] Zhang L, Zhao C, Ghimire B (2010). The role of concurrent chemoradiotherapy in the treatment of locoregionally advanced nasopharyngeal carcinoma among endemic population: a meta-analysis of the phase III randomized trials. BMC Cancer.

